# Cleaving the Halqeh-ye-nur diamonds: a dynamic fracture analysis

**DOI:** 10.1098/rsta.2014.0270

**Published:** 2015-03-28

**Authors:** Colin Atkinson, Philip M. Martineau, Rizwan U. A. Khan, John E. Field, David Fisher, Nick M. Davies, Julia V. Samartseva, Seth J. Putterman, Jonathan R. Hird

**Affiliations:** 1 Department of Mathematics, Imperial College London, London, UK; 2 Schlumberger Gould Research, Cambridge, UK; 3 DTC Research Centre, Maidenhead, Berkshire, UK; 4 Cavendish Laboratory, University of Cambridge, Cambridge, UK; 5 Department of Physics & Astronomy, University of California, Los Angeles, CA, USA

**Keywords:** diamond, cleavage, fracture

## Abstract

The degree of surface roughness and clarity with which a surface in a brittle material can be formed via fracture is known to be related to the speed of the propagating crack. Cracks traversing a brittle material at low speed produce very smooth surfaces, while those propagating faster create less reflective and rough surfaces (Buehler MJ, Gao H. 2006 *Nature* **439**, 307–310 (doi:10.1038/nature04408)). The elastic wave speeds (*c*_l_≈18 000 m s^−1^, *c*_*s*_≈11 750 m s^−1^) in diamond are fast (Willmott GR, Field JE. 2006 *Phil. Mag.* **86**, 4305–4318 (doi:10.1080/14786430500482336)) and present a particular problem in creating smooth surfaces during the cleaving of diamond—a routine operation in the fashioning of diamonds for gemstone purposes—as the waves are reflected from the boundaries of the material and can add a tensile component to the propagating crack tip causing the well-known cleavage steps observed on diamond surfaces (Field JE. 1971 *Contemp. Phys.* **12**, 1–31 (doi:10.1080/00107517108205103); Field JE. 1979 *Properties of diamond*, 1st edn, Academic Press; Wilks EM. 1958 *Phil. Mag.* **3**, 1074–1080 (doi:10.1080/14786435808237036)). Here we report an analysis of two diamonds, having large dimensions and high aspect ratio, which from a gemological analysis are shown to have been cleaved from the same 200 carat specimen. A methodology for their manufacture is calculated by an analysis of a model problem. This takes into account the effect of multiple reflections from the sample boundaries. It is suggested that the lapidary had an intuitive guide to how to apply the cleavage force in order to control the crack speed. In particular, it is shown that it is likely that this technique caused the fracture to propagate at a lower speed. The sacrifice of a large diamond with the intention of creating thin plates, rather than a faceted gemstone, demonstrates how symbolism and beliefs associated with gemstones have changed over the centuries (Harlow GE. 1998 *The nature of diamonds*, Cambridge University Press). The scientific insights gained by studying these gemstones suggest a method of producing macroscale atomically flat and stress-free surfaces on other brittle materials.

In the spring of 1645, the French traveller, merchant and jeweller Jean-Baptiste Tavernier, Baron of Aubonne, arrived at the Raolconda diamond mine in the present-day locality of Ramalakota, Andhra Pradesh, India. It was here that he noted adeptness in the art of cleaving diamonds exhibited by lapidaries employed in the vicinity of the mine workings. He observed that the flat surfaces obtained ‘qui sont de grande montre’ [make a great show] on account of the mirror-like reflections obtained on viewing them above the critical angle [1]. Here we describe a scientific examination of two thin diamonds of large surface area and high clarity that have been mounted in spectacle frames and which demonstrate these mirror-like reflections. Photographs of the individual diamonds together with their current setting are shown in figure 1.

**Figure 1.**
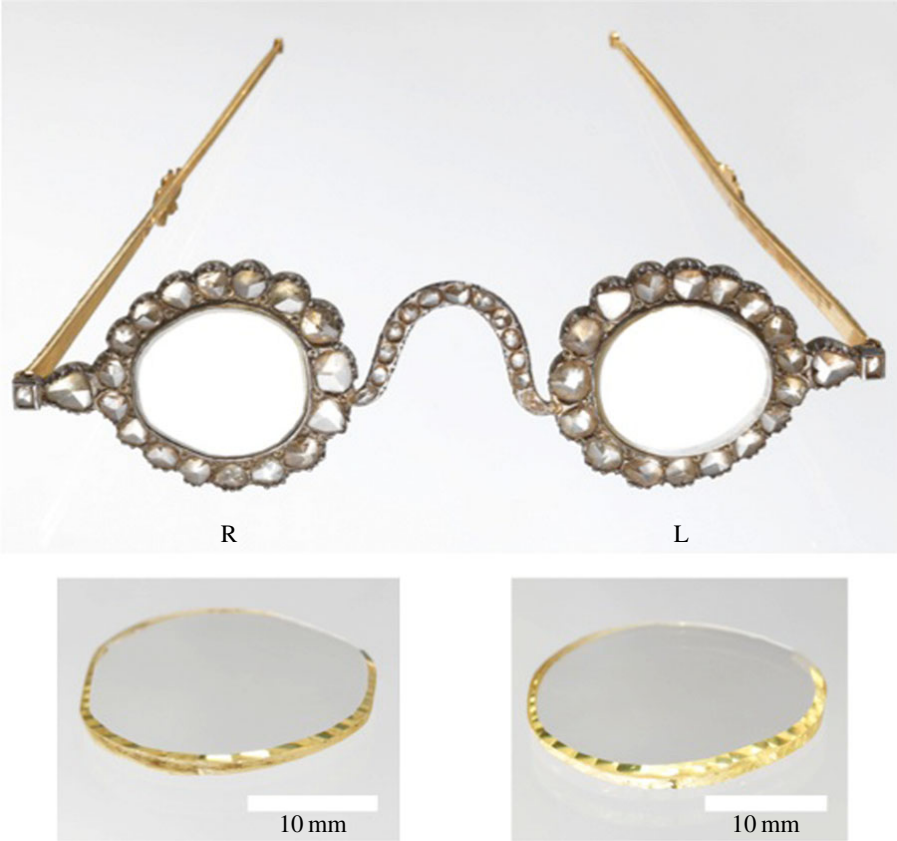
Photograph showing the current mounting of the diamond plates in spectacle frames (left, L and right, R according to wearer). The spectacle rims are surrounded by numerous diamonds of varying size. Inset: the diamonds removed from the frames. The mass of each stone is: right 2.299 g (11.495 carats) and left 2.563 g (12.815 carats). The diamond plates display a high degree of parallelism. The right plate has an average thickness of ≈1.59 mm while the left plate is ≈1.71 mm thick.

Scientifically, the diamonds are interesting for two principal reasons. First, the sectioning of any large natural diamond into thin plates is without precedent in modern times and provides a unique glimpse into the formation and growth of a large specimen of this most valued of gemstones. Second, the cutting and polishing of diamond into such thin specimens represents a technological challenge, especially as diamond is the hardest known material being highly brittle and being highly resistant to plastic deformation. These properties taken together with diamond's propensity to cleave along the octahedral {111} plane and a high thermal conductivity (1000–3500 W m^−1^ K^−1^) make it an unwieldy material to work, and suggest that the lapidary who created them was a highly skilled artisan well versed in the physical properties of diamond. Motivated by these reasons, we undertook both a gemological and mathematical analysis to attempt to describe the process by which the diamonds were wrought into their present forms.

In shape, the diamonds are roughly ovaloid having major and minor axes of *ca* 25 mm and 21 mm (left spectacle lens according to the wearer), respectively, and *ca* 24 mm and 23 mm (right) with both plates displaying a high degree of parallelism. The plates are thus not lenses in the optical sense; rather they are filters. The right plate is approximately 1.59 mm thick while the left is 1.71 mm thick. This gives both diamonds a high aspect ratio of ≈15:1. Both plates have been faceted at a low angle to the upper face around the perimeter in an irregular fashion.

From a gemological perspective, the stones appear flawless when examined with a loupe and appear colourless and transparent when viewed through the stone. The colour evident around the faceted edges suggests that they are in fact pale yellow.

A decision not to perform a study of the cathodoluminescence of the diamond was made because the possibility of damage could not be eliminated. However, viewing the diamonds in intense ultraviolet light reveals a spectacular growth history as shown in figure 2 which is unparalleled in terms of size and form. These fluorescence topographs were made using DiamondView^TM^, an instrument in which intense <230 nm ultraviolet radiation illuminates the surface of a diamond so that images of the resulting surface fluorescence can be recorded [2]. This instrument was originally developed to aid identification of synthetic diamond material using differences in characteristic growth structures relative to those of natural diamonds. For type IaAB diamonds, the fluorescence patterns are produced by variations in the nitrogen centre concentrations for material corresponding to different stages of the natural growth process. The patterning in these remarkable images is matched by the birefringence patterns [3,4] of the individual lenses (figure 3), which were recorded with a Wild M420 microscope equipped with crossed polarizers. Owing to the increased path length when they are observed edge-on, the diamonds exhibit a pale yellow colour indicative of type I natural diamond and this was confirmed by Fourier transform infrared (FTIR) spectra which were recorded at room temperature with a Nicolet Magna-IR 750 spectrometer with 0.5 cm^−1^ resolution. The UV–visible–near-infrared spectrum was recorded with a Perkin Elmer Lambda 1050 spectrometer with a data interval of 0.5 nm. Absorption spectroscopy of each diamond confirms the source of the yellow colour as being N3 nitrogen-related centres within the lattice. FTIR spectroscopy indicates that both diamonds contain both A and B nitrogen aggregates and therefore fall into the type IaAB category [5]. The atomic concentration of nitrogen in the A and B aggregates, [NA] and [NB], in each plate can be estimated [6,7]. For the left lens, [NA] is 578 ppm and [NB] 357 ppm, whereas for the right lens [NA] is 574 ppm and [NB] is 534.3 ppm. Both lenses are type IaAB and regular. The nitrogen contents and aggregation states are reasonably similar and are consistent with the lenses having been cut from the same stone which grew on average at slightly different times, but which experienced a similar thermal history. The difference in NB aggregates between the two lenses is thus due to sampling location.

**Figure 2.**
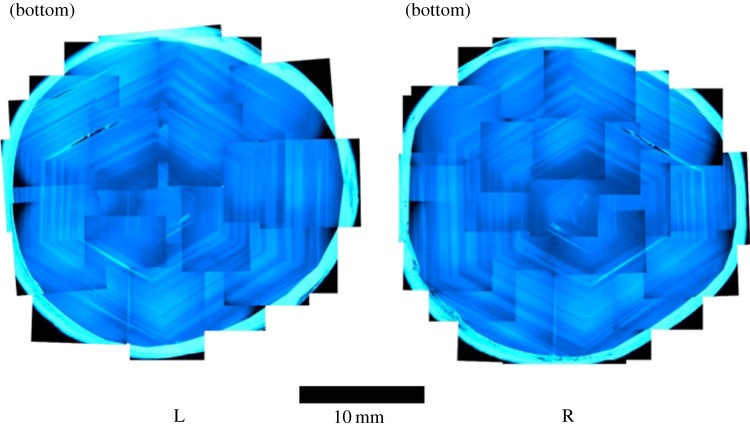
Fluorescence topographs of the two diamonds. The images are collages made from individual micrographs recorded using ultraviolet illumination in DiamondView^TM^. The contrast results from variations in the concentration of point defects for material corresponding to different stages of octahedral growth. The topographs are very close to being mirror images of each other. This suggests that the corresponding surfaces were originally coincident to one another in the same diamond and a cleave has been made to separate them. The fact that the patterns are close to regular hexagons is consistent with a final cleavage being made close to the centre of the stone.

**Figure 3.**
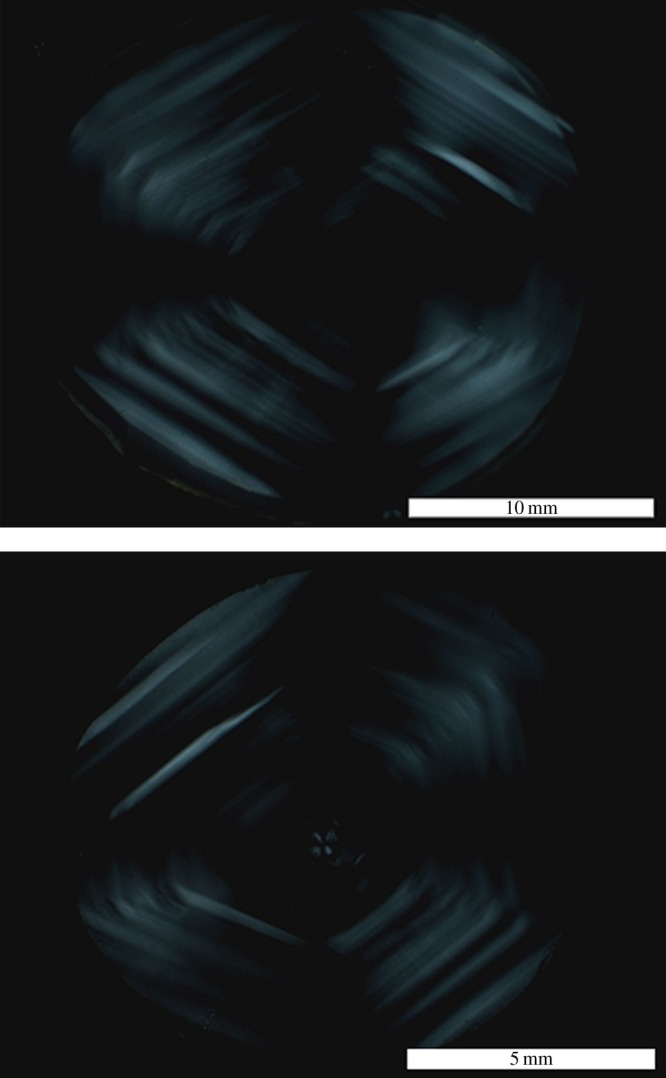
Birefringence micrographs of the diamonds recorded under crossed-polarizers oriented from left to right and from top to bottom of the images. Upper image is the left lens. Note the similarity of features shown here with the images taken under fluorescence (figure 2). (Online version in colour.)

The mirror-like symmetry observed in the fluorescence topographs and the birefringence show conclusively that the diamonds came from the same crystal from adjacent crystallographic planes; they are mating surfaces.

X-ray diffraction in the backscattering (Laue) geometry was carried out using a Photonic Science intensified Laue X-ray imaging camera system with a Philips PW1830 X-ray generator operating at 35 kV and 35 mA. The large facets of the crystal were aligned perpendicular to the X-ray beam and the observed spot patterns indicated that the large facets of both diamonds had orientations very close to {111}. With a detector–diamond distance of 31 mm and with the diamonds oriented with the 〈112〉 axis of the large facets aligned horizontally and perpendicular to the beam, software was used to simulate the observed spot patterns for both diamonds. If a diamond crystal is aligned with its [100] axis along the beam, [001] axis vertical and [010] orthogonal to these two directions, the following sequence of rotations of the crystal will result in the [111] being directly parallel to the beam: rotation by 45.0° about the beam direction, followed by rotation by 54.7° about the vertical. The required combination of rotations to simulate the observed spot pattern for the left diamond were found to be 44.3° about the beam direction, −0.1° about the horizontal direction perpendicular to the beam and −56.0° about the vertical.

The corresponding rotations required to simulate the observed spot pattern for the right diamond were 44.2°, −0.1° and −55.9°. This indicates that there is very little difference between the orientations of the large facets of the two diamonds.

By analysing the orientation, size and positioning of the two individual diamonds, the size of the original diamond from which they were manufactured can be estimated. This diamond weighed 200 carats—a large diamond by any standard. A measure of the parallelism was obtained by positioning the sample a few metres from a HeNe laser in such a way that the front surface reflection (in the centre of the sample) was returned along the in-coming laser beam. Then the deflection was measured for the laser spot corresponding to the weaker reflection from the back surface of the sample. The corresponding angle between the two faces was calculated using the tangent relationship. The sample was rotated to give wedge angles about each axis of the sample. Using this method, the left lens indicated a deviation from parallelism of 0.18°, the right lens 0.40°. Thickness variations measured using a micrometer indicated wedge angles consistent with these values. Optically, the surfaces of the diamonds are extremely smooth when viewed with the unaided eye and viewing above the critical angle yields a mirror finish. Nomarski microscopy of the diamond surfaces, however, shows that they have been well polished in distinct crystallographic directions (figure 4). This would have been a major technological challenge and demonstrates a high degree of craftsmanship. Diamond has a marked anisotropy in wear and friction which is present over an extreme range of tribological conditions of which the octahedral {111} plane exhibits the highest resistance of all planes to faceting [8,9]. It may, however, be polished (with difficulty) in 〈112〉 directions. Other surfaces of the diamonds show weaker polishing lines present in a direction 120° from the major polishing lines (which is also 〈112〉), suggesting that polishing took place in steps. In industry, a slight tilt of the octahedral plane towards the dodecahedral {110} plane is generally utilized in order to minimize damage to the polishing wheel (scaife), achieve acceptable wear rates and a good quality surface finish [10]. This technique has been used to polish the diamonds described in this paper. However, there is another further practical difficulty to polishing: breakage from mechanical and thermal shocks during the polishing process. The high aspect ratio of these diamonds means that breakage would have been a significant risk and suggests that the amount of polishing that took place was both minimal and carried out with a well-balanced polishing wheel. As both diamonds are characterized by a high degree of parallelism, similarity in thickness, and came from the same diamond the sequence of events which created these diamonds is of particular scientific interest.

**Figure 4.**
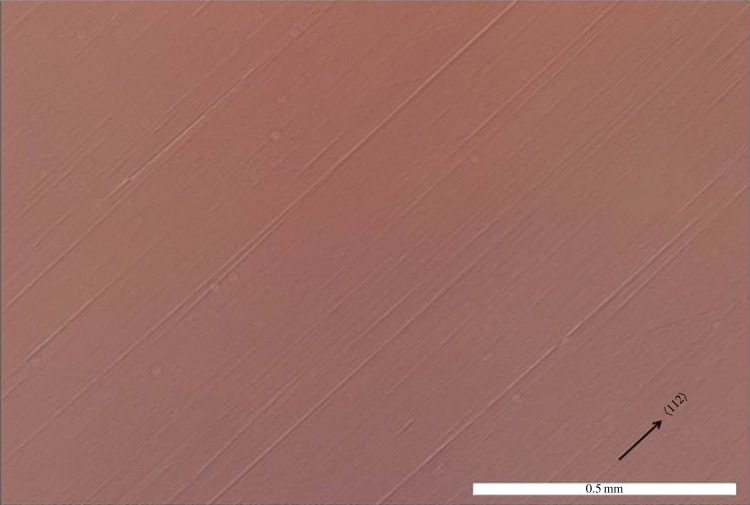
Microscopy of the diamond surfaces shows polishing relics consistent with conventional polishing directions used on octahedral {111} planes. Nomarski micrographs were recorded with an Olympus BX60 microscope. (Online version in colour.)

Two techniques could have been used to section the original stone into flat plates: cleaving or sawing. The technique of sawing of diamond was in its infancy until the late nineteenth century and consisted of a thin metal wire loaded with diamond powder and held in a bow which was dragged in a sawing motion along the plane to be cut—a laborious, time-consuming and tedious process [11]. The propensity of diamond to cleave along its octahedral plane (111), however, is undoubtedly one of the most ancient observations on the physical nature of diamond and is thought to be the process most likely used to form these plates.

In practice, cleaving is carried out by carefully scribing a V-shaped notch (kerf) of millimetre dimensions into the appropriate plane using a sharp-pointed diamond and requires a high degree of skill [12,10]. The diamond to be cleaved is inserted into a wooden stick and held in place with cement. The cleaver's blade (a knife of length 10 cm, height 5 cm and 0.3 cm thick tapered to blunt edge) is inserted into the kerf (the blade must not touch the bottom of the notch) and a sharp tap is given to this by means of a wooden or steel rod of 12 mm diameter [10]. The key to cleaving is to keep the fracture propagating in a straight line without branching on to other cleavage planes. Cracks in brittle isotropic solids such as glasses bifurcate when *v*/*c*_R_ (*v* is the fracture velocity and *c*_R_ is the Rayleigh wave velocity) exceeds ≈0.6. This velocity, the speed at which surface waves propagate on a stress-free surface, is the speed at which a propagating crack has an energy release rate which tends to zero (e.g. [13]). In solids with well-defined cleavage planes, including diamonds, this ratio, *v*/*c*_R_, approaches unity [14]. As noted above, the longitudinal elastic wave velocity *c*_l_ and transverse elastic wave velocity *c*_s_ are very high in diamond. During gem cleavage, the velocity of the growing crack *v* is significantly lower 1000–3000 m s^−1^ [14,15]. The stress wave thus travels ahead of the growing crack and will be reflected at an interface as the medium is finite. If *v*=3000 m s^−1^ and *c*_*l*_=18 000 m s^−1^ then for a wave reflecting off a boundary parallel to the cleavage plane a distance 1.7 mm away (the thickness of one lens) an interaction with the propagating crack will occur just 0.57 mm from the origin. Since the maximum length of the lenses is ≈26 mm (and the original crystal likely larger than this) the difficulty in forming the lenses is apparent. We model the cleavage process by assuming that a crack is produced that splits the specimen into two, the crack being driven by impacting the end of the specimen. We analyse this by means of a simple model in which the deformation is assumed to be that of longitudinal shear (this is similar to a model of the flint knapping process [16] except that in the flint knapping case there is only one stress free boundary). A blow which activates the cleavage launches a stress pulse into the specimen which interacts (and initiates) the moving crack tip. In this model problem, the block provides a displacement in the *z* direction which is antisymmetric about *y*=0 (figure 5). This can be envisaged as produced by a force −2*hf*′(*t*) at *x*<*x*_1_ uniformly spaced over each half of the specimen edge. The displacement (which must, of course, satisfy the wave equation) is thus
1
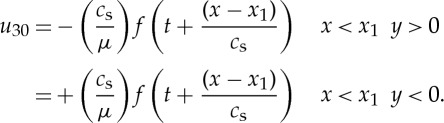

The notation here is that *u*_3_ is the displacement in the *z* direction and *u*_30_ is the field produced in the absence of the propagating crack. The cleaved plane shears on *y*=0. We suppose the end of the specimens is at *x*_1_=0. To simplify the analysis we assume the cut extends to the left so we have the displacement produced
2
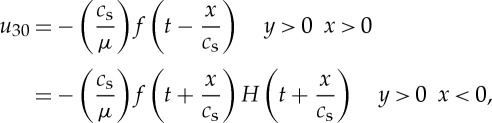

and the same with opposite sign for *y*<0. Here *c*_s_ is the shear wave speed and *μ* the shear modulus (a corresponding plane strain model would be somewhat more complicated) and *H* is the Heaviside step function. The specimen is assumed to have thickness 2*h* and to be much longer in the *z* direction. We assume that the crack starts to move with constant speed *v* when the pulse first hits it at *t*=0. If the head of the pulse has not yet reached the crack tip then *u*_30_ is given by equation (1). If, however, the solution (1) has evolved while the wave has overtaken the crack then the solution (1) will give a discontinuity in the mode 3 displacement *u*_30_ ahead of the crack. This must be taken into account in the analysis of the problem. The solution to the problem requires writing the displacement as *u*_3_=*u*_30_+*u*_3_′ where *u*_3_′ has to be found to correct for this discontinuity leaving the total displacement and stress continuous ahead of the crack; the crack being, of course, stress free. For our model problem this requires solving the wave equation in the displacement *u*_3_ with shear wave speed *c*_s_ subject to stress free boundary conditions on the sides of the sample (at *y*=±*h*) and a stress free crack running at speed *v* on *y*=0. If we use a coordinate *x*′=*x*−*vt* moving with the crack tip which moves in the strip *y*=±*h* whose sides are traction-free then we find that the Laplace transform of the stress intensity factor at the crack tip is
3
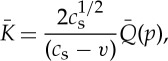

where *p* is the Laplace transform variable and the energy release rate into the crack tip will be
4
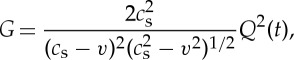

where *Q*(*t*) is the inverse of 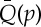 with
5
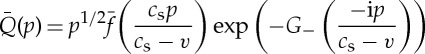

and
6
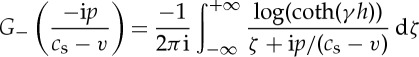

with
7
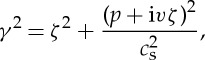

where *ζ* is the Fourier transform variable used in the analysis.

**Figure 5.**
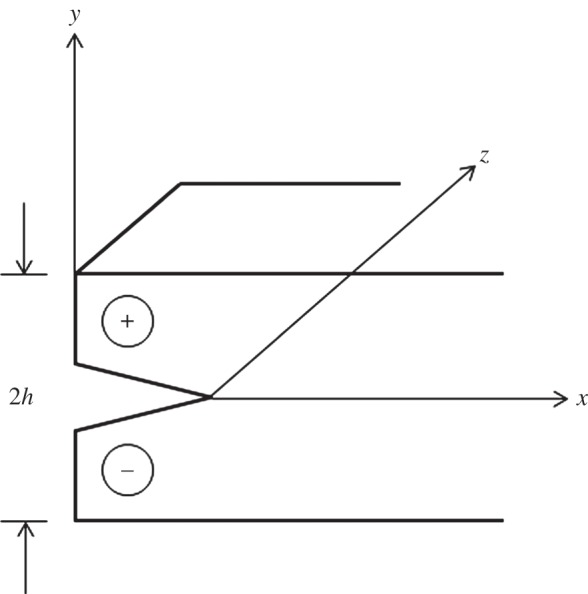
Schematic of a crack tip traversing a thin specimen of thickness 2*h* subject to the boundary constraints: −*h*<*y*<*h*, *u*(*x*,*y*,*z*)=(*u*_1_,*u*_2_,*u*_3_).

These formulae are derived with the boundary condition that the sides *y*=±*h* are stress free. To obtain values for *Q*(*t*) and hence of the energy release rate, we are required to numerically evaluate the function *G*_−_ and the inverse of 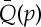, *p* being the Laplace transform variable. From the above expressions, we can show that the energy flow *G* into the moving crack tip [13] is proportional to
8
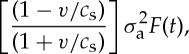

where *F*(*t*) is given by numerically evaluating complex integrals. We work with a variable *t*_1_ defined as
9
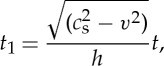

and *t*_c_ the time for a constant speed crack of velocity *v* to traverse the specimen. For a specimen of length 30 mm and half thickness 1.77 mm, *t*_c_=30/*αc*_s_ with *v*=*αc*_s_ and *c*_s_=11.6 mm μs^−1^. Thus,
10
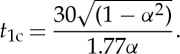

Note *α*=*v*/*c*_s_≤0.99311 implies many reflections from the sides *y*=±*h*. We plot the curves of *σ*_a_, the magnitude of the applied force against position as the crack traverses the sample, in figure 6 (the relative times at which the crack reaches each position are, of course, proportional to *α*). The results plotted here have assumed a suddenly applied force of magnitude *σ*_a_ distributed uniformly across the sample edges. The material is assumed to have a fixed *G*_c_ (the critical value at which fracture occurs) and the curves show the variation of *σ*_a_ with time for different velocities. As can be seen, the variation in *σ*_a_ is least for the smaller crack velocities even though the analysis has assumed *σ*_a_ constant. We might expect that the lapidary re-adjusts *σ*_a_ (i.e. the force profile) based on experience in selection and placement of the blade, rod and holding tools, and their compliance and skill in delivering the impulse of the initial blow. These all combine to impart a force profile that achieves smooth fracture. This would, of course, be an extremely sensitive matter and a reflection of the skill of the lapidary. This would be more likely at the lower crack speeds as it is precisely at these speeds that cracks create atomically flat mirror-like surfaces. At higher speeds, rougher, less reflective (mist) and finally very rough surfaces are formed [17].

**Figure 6.**
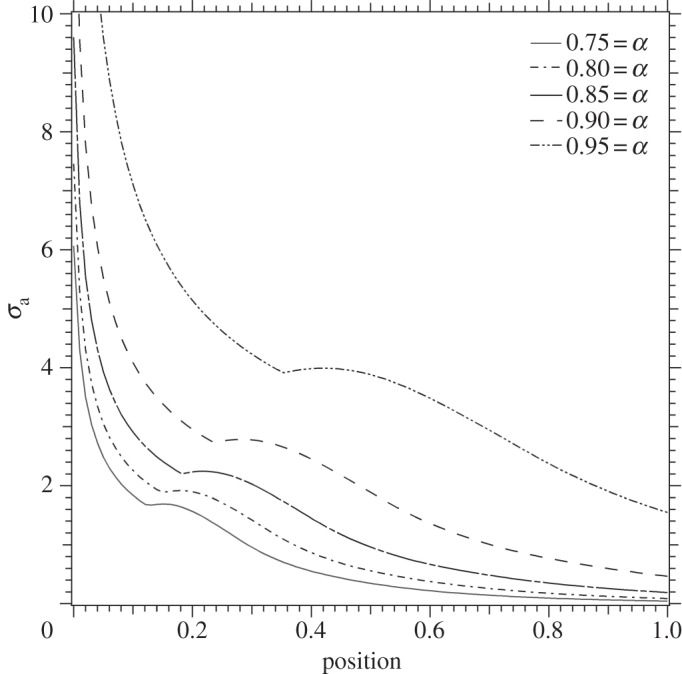
The variation in *σ*_a_, the magnitude of the applied force, plotted against position for different velocities (i.e. for different values of *α*), as the crack traverses the sample from 0 to 1.

It should be noted that our analysis takes into account multiple reflections from the sides of the sample. In particular, the effect of the first reflection is shown as a distinctive kink in the curves in figure 6. As *α* decreases, more reflections are taken into account. The analysis described above is intended to represent the final cleave where the two plates are separated from each other as illustrated in figure 7*d*. We conjecture that a total of three cleaves would have been necessary to section the original diamond into the current morphology. To achieve the morphology illustrated in figure 7*c* from that shown in figure 7*b*, a process similar to that used in percussive flint knapping would have been used [16]. Creating the morphology shown in figure 7*b* from the original crystal, figure 7*a*, would have been a similar scenario to the analysis described in this paper. Well-cleaved diamonds are typically characterized by steps of between 10 and 200 nm in height [18] and are believed to be Wallner lines [19] created by the crack tip interacting with crystallographic imperfections and interactions between reflected waves and the crack tip. It would have been these that were removed by polishing, forming distinctive polishing lines on the surfaces of the plates. The final step of processing would have been the faceting around the edges of the diamonds. Unfortunately, there is no known method of locating the geological source of the diamonds described here. The large size of the original stone and the current morphology, however, are suggestive of an Indian origin. Tavernier's account of cleaving in India is the first accurate description of the cleaving process to form large facets on gemstones [1] and the fashion for flat diamonds used for decorative purposes continued in India until the early half of the twentieth century [20]. Mystical properties of gemstones still have their allure in the Indian subcontinent and are described in lapidary works such as the Ratnaparîkṣâ [21]. Whatever their original purpose, mounted as spectacle ‘lenses’ they are a singularly impressive and eccentric example of a trait which also has historical precedent: Pliny's Natural History recounts Emperor Nero observing gladiatorial contests through, or off the surface of emeralds [22]. The soothing nature of the emerald to the eye, and its supposed medicinal benefits for all manner of ailments is a common theme in both European and Indian lapidaries [23] and an inventory of the treasury during the reign of Charles V of France lists beryls framed as spectacles [24].

**Figure 7.**
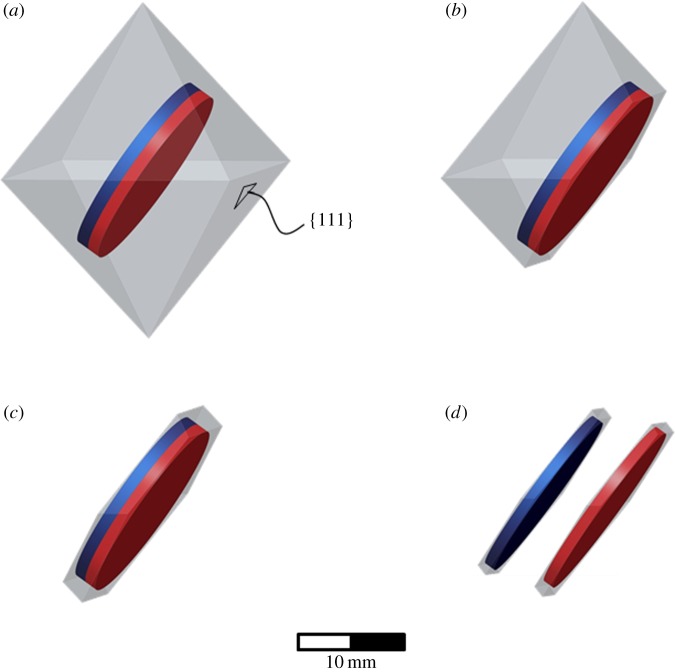
(*a*) A visualization of the diamond plates, coloured red and blue, contained within a large diamond of octahedral habit. The minimum weight of the original crystal would have been above 40 g (200 carats). (*b*–*d*) The changing morphology of the original crystal as it was cleaved sequentially. At each stage a kerf would need to be inscribed into the diamond to help initiate the crack. The plate illustrated in (*c*) is ≈3.4 mm thick (twice the final plate thickness), 33 mm long and 23 mm in width. This was subsequently cleaved to make the two thin plates shown in (*d*). Note that additional processing would have been needed in stage (*c*) as there is no room for a kerf if the points are not first ground down. After the final cleave, polishing of the flat plates and faceting of the edges were carried out.

We have given a plausible explanation as to how a lapidary has produced two thin diamonds of high aspect ratio. Our model analysis could, in principle, be extended to a full three-dimensional analysis of the cleavage process taking into account the multiplicity of wave reflections which may occur.
